# Experimental Beetle Metapopulations Respond Positively to Dynamic Landscapes and Reduced Connectivity

**DOI:** 10.1371/journal.pone.0034518

**Published:** 2012-04-03

**Authors:** Byju N. Govindan, Robert K. Swihart

**Affiliations:** Department of Forestry and Natural Resources, Purdue University, West Lafayette, Indiana, United States of America; University Copenhagen, Denmark

## Abstract

Interactive effects of multiple environmental factors on metapopulation dynamics have received scant attention. We designed a laboratory study to test hypotheses regarding interactive effects of factors affecting the metapopulation dynamics of red flour beetle, *Tribolium castaneum*. Within a four-patch landscape we modified resource level (constant and diminishing), patch connectivity (high and low) and patch configuration (static and dynamic) to conduct a 2^3^ factorial experiment, consisting of 8 metapopulations, each with 3 replicates. For comparison, two control populations consisting of isolated and static subpopulations were provided with resources at constant or diminishing levels. Longitudinal data from 22 tri-weekly counts of beetle abundance were analyzed using Bayesian Poisson generalized linear mixed models to estimate additive and interactive effects of factors affecting abundance. Constant resource levels, low connectivity and dynamic patches yielded greater levels of adult beetle abundance. For a given resource level, frequency of colonization exceeded extinction in landscapes with dynamic patches when connectivity was low, thereby promoting greater patch occupancy. Negative density dependence of pupae on adults occurred and was stronger in landscapes with low connectivity and constant resources; these metapopulations also demonstrated greatest stability. Metapopulations in control landscapes went extinct quickly, denoting lower persistence than comparable landscapes with low connectivity. When landscape carrying capacity was constant, habitat destruction coupled with low connectivity created asynchronous local dynamics and refugia within which cannibalism of pupae was reduced. Increasing connectivity may be counter-productive and habitat destruction/recreation may be beneficial to species in some contexts.

## Introduction

Metapopulations are local populations distributed patchily in space and linked by dispersal [1]. Their viability depends on a variety of habitat and species-specific features. Models predict that habitat characteristics such as amount [2], [3], suitability [2], [4], [5], spatial structure [6], [7], and connectivity [8], [9] are important determinants of extinction-colonization dynamics and hence metapopulation persistence. The spatial [10], [11] and temporal [12] dynamics in the availability of habitable and unsuitable habitats also are predicted to have important consequences for metapopulation dynamics. Unfortunately, few studies have simultaneously explored effects of multiple factors on metapopulation dynamics. Our objective was to test how resource availability, patch connectivity, and dynamics of patch configuration interact to influence metapopulations.

Numerous prior studies have examined the role of each of these factors separately. Level of resource availability (often measured using patch area or quality) has emerged predictably as an important determinant of metapopulation viability [13]. Resource loss can result from either gradual depletion of resources from a patch or outright destruction of patches. Gradual depletion of resources from a patch, while reducing carrying capacity, does not alter the connectivity between patches in a landscape. Rather, gradual depletion can induce higher adult dispersal and mortality and lower reproduction, while increasing immature mortality and development time [14]. In contrast, rapid destruction of a patch in a landscape reduces the number of habitats available for occupancy, increases inter-patch distances, decreases connectivity of resource patches, and can lead to rapid extinction beyond a critical threshold of loss [14], [15].

Reduced connectivity of resource patches has lowered persistence for metapopulations of fruit flies (*Drosophila hydei*) [16]. However, the relation between connectivity and persistence is not always monotonic, as intermediate levels of connectivity enhanced persistence for other metapopulations [17], [18]. Moreover, if local extinction rate covaries with connectivity or dispersal rate, an anti-rescue effect may lead to reduced stability and persistence by, e.g., facilitating the spread of contagious disease between subpopulations, enhancing predation pressure, or synchronizing local population dynamics [9], [17], [19]–[22].

In dynamic landscapes, i.e., landscapes in which patches are destroyed and re-created over time, disturbances that render patches unsuitable increase local extinction and reduce the number of empty habitats available for colonization [23]. Alternatively, patches that are less prone to destruction can serve as refugia and a source of colonists, thereby enhancing metapopulation persistence [24].

We manipulated resource availability, patch connectivity, and dynamics of patch configuration in experimental metapopulations to investigate their additive and interactive effects. Specifically, we tested these effects by manipulating the amount of resources and the level of boundary permeability [25], [26] for red flour beetles (*Tribolium castaeneum* Herbst (Coleoptera: Tenebrionidae)). We tested the predicted main effects summarized in the preceding paragraphs and examined all pairwise interactions of these effects on colonization, extinction, and abundance of beetles.

## Methods

### Landscapes and experimental design

Red flour beetle is a stored grain pest that infests a variety of stored products worldwide. The stock population (Berlin) of *Tribolium castaneum* was obtained from the U.S. Grain Marketing Research Laboratory in Manhattan, Kansas, in 2005. Beetles were cultured in 95% wheat flour and 5% yeast medium by mass. Beetles were maintained in an environmental chamber at 33±1°C and 70±5% relative humidity. The life cycle in *T. castaneum* (egg to adult) takes roughly one month, with an average 4 days for egg, 3 weeks for larval and 6 days for pupal development [27]. Adults attain sexual maturity and start laying eggs in 2–3 days of emergence [28]. Thus, the duration of our experiment (23 tri-weekly period) corresponded to 14–16 generations [13].

#### Constructed landscapes

**Figure 1:**
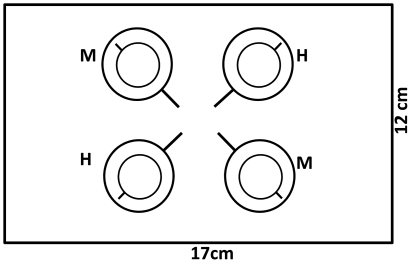
Schematic representation of experimental landscapes consisting of two patches of habitat (H, 95% flour and 5% yeast by mass) and two patches of marginal habitat (M, dextrose). Each patch consisted of an inner and outer Petri dish, with resources contained in the inner one. The dark lines projecting from the outer and inner Petri dish denote the paper ramps for dispersing beetles. A small hole on the rim of the outer Petri dish beneath the point where each paper ramp is attached served as an exit hole. Dimensions of box and patches are not to scale.

We designed experimental landscapes with two habitat and two “marginal” habitat patches, arranged in an alternating sequence (Fig. 1). Habitat patches consisted of 95% wheat flour and 5% brewer's yeast by mass. Preliminary studies demonstrated that this mixture provided a resource that favored the successful reproduction and survival of the beetles. “Marginal” habitat patches consisted of powdered cane sugar (dextrose). Preliminary studies revealed that the dextrose medium prevented successful reproduction but permitted adult survival [13].

Each constructed landscape consisted (see Fig. 1) of a 17 cm×12 cm plastic box (Pioneer Plastics, Dixon, KY). The floor was painted with white pigmented primer sealer (William Zinsser and Co., www.zinsser.com) containing fine-grained sand to facilitate beetle traction. To confine insects, the sides of the tray were treated with Fluon (Northern Products Inc., Woonsocket RI). A patch in a landscape consisted of a small (35 mm diameter×10 mm height) Petri dish affixed with glue inside the center of a larger (60×15 mm) Petri dish. Gluing was done all along the rim of the smaller Petri dish to seal the base and prevent adults or larvae from crawling under the smaller dish and thus getting trapped over the course of the experiment. To facilitate beetle movement between the inner and outer portions of the patch, an inverted-V paper ramp (24×4 mm) was attached to the rim of the inner dish. The lid of the inner small Petri dish was notched where the inverted-V paper ramp joined the inner Petri dish to allow the exit of beetles to the outer Petri dish. A circular hole of diameter 2 mm was made in the outer dish and oriented 180 degrees from the inverted paper ramp of the inner Petri dish to allow emigration of beetles from a patch into the surrounding landscape. Connectivity, specifically, patch boundary permeability [5], [25], [26], was varied by modifying the height at which these exit holes were placed. Preliminary trials over a 3-week period demonstrated that patches with holes 2.5 mm above the base of the outer dish exhibited emigration rates 5.8 times greater than patches with exit holes at a height of 4.0 mm. The entry of beetles back into patches was facilitated by providing paper ramps (22×4 mm) attached to the edge of the outer dish. The exit holes and entry ramps that facilitated the movement of the beetles into and out of the surrounding landscape, respectively, were positioned in a small circular area at the center of the landscape, thereby assuring comparable distances to all other patches and reducing the effects associated with beetles wandering along the edges of the landscape. Resources were placed inside the smaller dish, which could hold a maximum of 3 g of medium. Keeping the resources concentrated in the interior of the smaller patch minimized spillover of resources into the matrix. In addition, restricting resources in the interior dish maintained the exit hole on the outer dish at a constant height.

#### Initial conditions and data collection

All experimental patches received 6 adults and 18 larvae released on 3 g of medium (flour in habitats and dextrose in marginal habitats) inside the small dish of each patch (total = 24 adults and 72 larvae per landscape). Both adults and larvae were added as a starter population to mimic more closely established local populations, avoid time lags, and buffer against crashes associated with density-dependent cannibalism [14]. Initial population sizes were chosen to approximate the maximal carrying capacity of the landscape, based on preliminary trials. Twenty four adults were used per landscape, even though estimated carrying capacity was 16, to increase the likelihood that all life stages were equally distributed in the 4 patches and to increase odds of 1∶1 sex ratios. We observed a sex ratio of 1∶1 when sex of 100 random pupae was determined (unpublished data). For a sample of six individuals, the probability of obtaining all adults of a single sex in a patch is 0.03, whereas the probability of 2–4 adults of a given sex is 0.78. For larvae, the probability of obtaining 18 individuals of the same sex is 7.6×10^−6^, and the probability of 7–12 larvae of a given sex is 0.90.

For each treatment, observations were made every 3 weeks, a time interval chosen to correspond with the larval developmental period, allow sufficient time for beetles to respond to the treatments imposed, and minimize the disturbance associated with counting. Sifting the resources with #80 mesh sieve retains most of the *Tribolium* eggs in the medium [29]. Measurements were made by sifting the resources using #20 and #80 mesh sieves and counting the number of living larvae, pupae and live and dead adults in the patches and the surrounding matrix outside the patches. All living individuals in all life stages were returned to the patch they had occupied during the most recent count. Live beetles in the matrix also were counted and released back into the matrix. Experiments were continued for 22 3-week observation periods or until metapopulation extinction.

#### Experimental design

We used a factorial design including 3 factors, each at 2 levels, for a total of eight treatments. Each treatment was replicated three times. The factors were landscape connectivity, resource level, and patch configuration. Connectivity was manipulated via high (exit holes at 2.5 mm) and low (holes at 4.0 mm) boundary permeability. Resource levels of landscapes either remained constant throughout the experiment, i.e., the medium in each patch was replenished every 3 weeks, or diminished to represent habitat degradation. For the latter treatment, at the end of every 3-week period, the medium in habitat and marginal-habitat patches was replaced with fresh medium, but in an amount reduced by 0.5 g from what had been present in the patch 3 weeks earlier. For instance, at the end of the first 3 weeks, a habitat patch with 3 g of resource was replenished with 2.5 g of fresh resource and similarly, a marginal-habitat patch was replenished with 2.5 g of dextrose. Reduction of patch resources by 0.5 g every 3 weeks was continued until the total resource available in a patch was reduced to 0.5 g, at which point a final reduction to 0.2 g was made. Below this resource level cannibalism is quite high [30]. Patch configuration was manipulated either by maintaining a fixed identity of habitat (flour) and marginal-habitat (dextrose) patches for the duration of the experiment (static), or destroying all habitats and restoring all marginal habitats to habitat status at tri-weekly intervals (dynamic). For dynamic landscape treatments, the entire contents of both habitat patches were removed, and all stages of beetles were sieved and counted. Next, habitat patches were “destroyed”, i.e., converted to marginal-habitat patches containing dextrose medium. Similarly, marginal-habitat dextrose patches were restored to habitat patches containing flour medium. Beetles then were returned to the patch from which they had been counted and whose state had changed (e.g., from habitat to marginal-habitat). This pattern of habitat destruction and restoration mimics rotational cropping systems of many agro-ecosystems and incorporated temporal dynamics in configuration of patches while maintaining a constant landscape capacity from a resource perspective.

In addition to the 2^3^ factorial experiments, we included as references two controls with no landscape connectivity, i.e., no dispersal of beetles from static patches. In one control, carrying capacity remained constant, whereas in the other carrying capacity diminished over time as described above. Only static patch configurations were used in control landscapes, because extinction would be inevitable in landscapes with dynamic patch configuration (i.e., destruction of habitable patches) and no dispersal. Each control was replicated three times.

### Statistical analysis

#### Colonization and extinction

Probabilities of colonization and extinction for each patch state (habitat or marginal habitat) were calculated as the proportion of those colonization and extinction events occurring from time t-1 to time t during 22 tri-weekly surveys (except as noted below) divided by the total number of patches in a particular state and available for colonization (i.e., unoccupied) or extinction (occupied) respectively at time t-1. For analyses involving comparison across landscapes with diminishing resources or controls, only data from the first 18 tri-weekly surveys were used, because metapopulations in all three replicate landscapes suffered extinction beyond this time.

Patch extinction was defined as absence of adult beetles at time t after being occupied by adults at time t-1. Conversely, patch colonization was defined as the presence of at least one adult in a patch following extinction. Data on colonization and extinction frequencies in both patch states of each replicate landscape were used to derive mean colonization and extinction probabilities at the landscape level for each treatment. Because count data from the experiment were overdispersed, a quasi-binomial generalized linear model was fitted [31] to the proportion of successful colonization and extinction events of each landscape. Predicted coefficients on a logit scale were back transformed to proportions for comparison. For all analyses, independent variables included level of resource (constant = 1, diminishing = 0), connectivity between patches (high = 1, low = 0) and patch configuration (static = 1, dynamic = 0). Model selection was conducted using the quasi Akaike Information Criterion (QAIC) [32] to account for overdispersion.

#### Metapopulation dynamics

We applied a generalized linear mixed effects Poisson model (GLMM) to determine how resource level, patch connectivity and patch configuration affected metapopulation attributes. The GLMM was implemented within a Bayesian framework [33]. Specifically, the response trajectory of each landscape was modeled as a mixture of the population responses shared by all landscapes (fixed effects) and effects unique to each individual landscape (random effects), enabling us to account for over-dispersion.

Response variables in the GLMMs included total live adults, live adults inside patches, adults outside patches, larvae inside patches, and pupae inside patches. We fitted a repeated measures model with all main and two-way fixed effects and two random effects using the model:
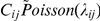


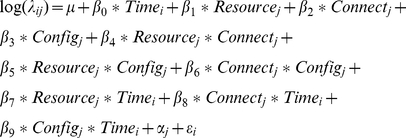
In the model, *C_ij_* is the count observed in landscape *j* (*j* = 1 to 24) at time step *i* (*i* = 1 to 22). The intercept *μ* represents the grand mean effect, and *β*s are the coefficients associated with fixed effects. The parameters *α_j_* and *ε_i_* account for random variation in beetle count data due to landscape and time effects, respectively. Uninformative normal priors with mean zero were used for *μ*, *β*, *α_j_* and *ε_i_*. Standard deviations of 100 were specified for the fixed effects parameters, whereas the hyperparameters *σ_α_* and *σ_ε_* reflect the random variation due to landscapes and time, respectively, and were drawn from a Uniform (0, 1) distribution.

Our control landscapes lacked connectivity between patches. To model the effect of complete isolation on the number of live adults in patches, we modified the GLMM described above to include three levels of connectivity (none = 0, low = 1, high = 2) and to exclude main and interaction effects associated with patch configuration. We also fitted a Poisson GLMM to assess the nature of density dependence on pupae and larvae at time t in patches of control and treatment populations. For this analysis, time and number of live adult beetles in patches at time t-1 were treated as fixed factors, along with random landscape and time effects.

The GLMMs were fitted by calling WinBUGS 1.4 [34] directly from free software package R version 2.9.2 [31] using the R add-on library R2WinBUGS [31]. For each fitted model, three parallel chains were run, each with 40000 iterations and a thinning rate of 35, discarding the first 5000 iterations as burn-in. Gelman-Rubin R-hat values (< = 1.1) were used to assess convergence of chains [35]. Our WinBUGS script is provided as a supplement (Appendix S1).

#### Metapopulation stability

We estimated stability of metapopulations by measuring the mean amplitude of fluctuations in population size over time. Specifically, we computed a fluctuation index [17] representing the mean change in population size from t to t+1, scaled by average population size over the duration of the study. We estimated fluctuation indices for all metapopulations and each of the associated subpopulations and performed an ANOVA on the fluctuation indices to investigate the main and interaction effects of resource level, connectivity and patch configuration on metapopulation stability. We also performed an ANOVA on the fluctuation indices estimated for subpopulations in habitat and marginal habitat with the same set of predictor variables.

## Results

### Colonization and extinction

**Figure 2:**
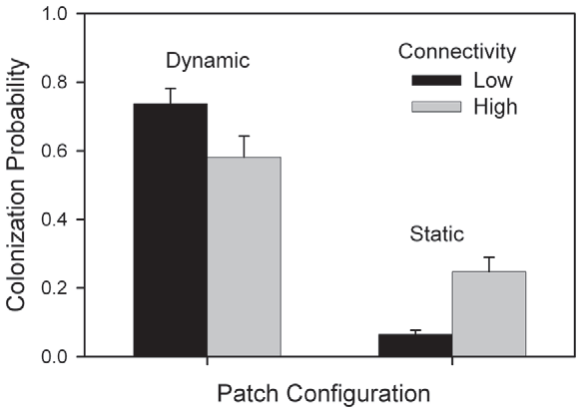
Increased connectivity dampened colonization differences between dynamic and static landscapes. In landscapes with low patch connectivity, frequency of mean colonization was 9 times higher when patches were dynamic than static. In high-connectivity landscapes, mean colonization frequency was only 2.5 times higher in dynamic than static landscapes.

For colonization frequency, the QAIC-best model included a significant (P = 0.0003) interaction effect of patch connectivity and configuration. Increased connectivity dampened the positive effect of a dynamic patch configuration on colonization (Fig. 2). Specifically, colonization frequency (and probability) in landscapes with low patch connectivity was nearly 10 times greater when patches had dynamic versus static configuration. In contrast, colonization frequency (probability) in landscapes with high patch connectivity was only 2.5 times higher when patches were dynamic versus static.

The frequency of patch extinctions was 1.65 times greater for landscapes with diminishing resources (P = 0.01) and nearly three times greater in landscapes with dynamic patches (P≪0.001). No interactive effects on patch extinction were observed.

At the landscape level, colonization probabilities were on average 2.6 times greater when resource was constant versus diminishing (P≪0.0001) and 4.9 times greater when patch configuration was dynamic versus static (P≪0.0001) (Table 1). Ratios of colonization∶extinction were highest for landscapes with constant resources and dynamic patch configuration (Table 1).

**Table 1:** Colonization and extinction probabilities at the patch and landscape level for all metapopulations in the 2^3^ factorial experiment.

Trtmt #	Factors			Colonization Probability (C)			Extinction Probability (E)			C/E
	Connectivity	Resource	Configuration	Marginal Habitats	Habitats	Landscape	Marginal Habitats	Habitats	Landscape	Landscape
1	High	Constant	Static	0.34	*	0.31	0.61	0.02	0.17	1.88
2	High	Constant	Dynamic	*	1.00	1.00	0.56	0.02	0.40	2.52
3	High	Diminish	Static	0.08	0.25	0.13	0.80	0.22	0.30	0.42
4	High	Diminish	Dynamic	0.00	0.37	0.24	0.74	0.08	0.58	0.41
5	Low	Constant	Static	0.12	*	0.12	0.90	0.00	0.12	1.05
6	Low	Constant	Dynamic	*	1.00	1.00	0.55	0.00	0.38	2.64
7	Low	Diminish	Static	0.02	0.00	0.01	0.56	0.08	0.15	0.08
8	Low	Diminish	Dynamic	0.00	0.68	0.56	0.82	0.02	0.68	0.82
C1	None	Constant	Static	0.00	0.00	0.00	1.00	1.00	1.00	0.00
C2	None	Diminish	Static	0.00	0.00	0.00	1.00	1.00	1.00	0.00

Estimates were based on 22 and 18 tri-weekly surveys for constant- and diminishing-resource landscapes, respectively. Asterisks indicate a lack of colonization events. C1 and C2 denote controls and thus lacked colonization and always went extinct.

### Metapopulation dynamics

Abundance of one or more beetle life stages was influenced by the main effects of resource level, patch connectivity, and patch configuration. Not surprisingly, resource level was the most influential factor affecting abundance of all life stages, with standardized coefficients that were 1.7–33.8 times larger than the next most influential main effect (Table 2, 3). Abundance of each of the beetle life stages also was affected substantially by pairwise interactions of two or more main effects. The magnitude of standardized coefficients for significant pairwise interactions of resource level, patch connectivity, and patch configuration averaged 16% of the corresponding coefficient for resource level (Table 2).

**Table 2:** Estimates of parameters (β) and 95% credible intervals for Poisson mixed effects regressions of adult, larval and pupal counts.

Fixed Effect Parameters	TLA	TAO	Larvae	Pupae
Intercept	2.79 (2.6–2.97)*	1.75 (1.28–2.20)*	3.85 (3.63–4.06)*	0.66 (0.05–1.24) *
Time	−0.28 (−0.34–−0.21)*	0.08 (−0.26–0.42)	−0.06 (−0.15–0.03)	0.41 (0.04–0.78) *
Resource	1.70 (1.45–1.95)*	2.41 (1.94–2.91)*	2.03 (1.76–2.29)*	2.59 (1.86–3.34) *
Connectivity	−0.02 (−0.27–0.22)	1.39 (0.94–1.89)*	0.04 (−0.21–0.29)	0.22 (−0.40–0.83)
Configuration	−0.14 (−0.37–0.09)^a^	−0.10 (−0.54–0.36)	0.01 (−0.24–0.28)	0.05 (−0.59–0.68)
Resource×Connect	0.17 (−0.10–0.44)^b^	−0.04 (−0.61–0.54)	0.14 (−0.18–0.43)	0.18 (−0.62–0.98)
Resource×Config	−0.21 (−0.49–0.06)^c^	0.04 (−0.52–0.61)	−0.20 (−0.50–0.10)^e^	−0.27 (−1.10–0.52)
Connect×Config	0.14 (−0.15–0.41)	0.59 (0.06–1.16)*	0.19 (−0.09–0.49)^f^	0.73 (0.03–1.54)*
Time×Resource	1.11 (1.03–1.18)*	1.64 (1.43–1.87)*	1.34 (1.29–1.40)*	1.40 (1.07–1.75)*
Time×Connect	−0.12 (−0.17–−0.06)*	0.01 (−0.13–0.14)	0.01 (−0.03–0.04)^g^	0.21 (0.05–0.37)*
Time×Config	−0.05 (−0.11–0)^d^	−0.02 (−0.13–0.10)	0.08 (0.05–0.12)*	0.18 (0.02–0.35)*

Data on response variables were collected over 23 3–week periods. Response variable ‘TLA’ stands for total live adults in landscape, and ‘TAO’ for total adults outside patches in landscape. An asterisk indicates that the 95% credible interval did not contain zero. Lower-case superscripts are provided for interaction effects with credible intervals containing zero but for which only a small fraction, *f*, of the posterior distribution was more extreme than zero.

a *f* = 0.11;

b *f* = 0.10;

c *f* = 0.06;

d *f* = 0.02;

e *f* = 0.08;

f *f* = 0.11;

g *f* = 0.07.

**Table 3:** Mixed effects Poisson regression estimates and 95% credible intervals for landscapes with static patches and control landscapes (with no connectivity).

Dependent Variable	Parameter	β	95% CI	
			Lower	Upper
Live adults in patches	Intercept*	2.57	2.39	2.75
	Time*	−0.24	−0.32	−0.16
	Connectivity^a^	0.12	−0.09	0.32
	Resource*	1.26	1.00	1.52
	Time×Resource*	0.91	0.81	1.01
	Time×Connect*	0.32	0.26	0.37
	Connect×Resource	0.02	−0.28	0.31

For interpretation of *, see footnote for Table 2.

a The fraction of the posterior distribution more extreme than zero, *f* = 0.13.

**Figure 3:**
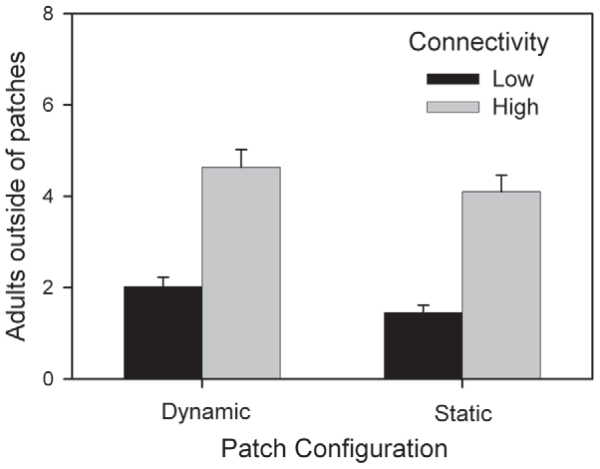
The number of adult beetles found in the matrix outside of patches was positively affected by high connectivity and dynamic patches. Mean (+SE) abundance in the matrix outside of patches was greatest when connectivity was high and when patches were dynamic. The ratio of abundance in high and low connectivity treatments (N_high_∶N_low_) was 4.1∶1.4 for static and 4.6∶2.0 for dynamic configuration, or a 22% greater effect of high connectivity with static configuration relative to dynamic configuration.

Dynamics of adult beetles were affected by interactions of time with each of the experimental variables, and by interactions of resource×connectivity and resource×patch configuration (Table 2). Adult abundance in landscapes declined over time, with more rapid declines for populations characterized by diminishing (versus constant) resource levels, high (versus low) connectivity, or static (versus dynamic) patch configuration. Total adult abundance averaged 2.8 times higher in landscapes with constant resources, and this effect was slightly lower (8%) in landscapes with low connectivity. Effects of patch configuration were evident only in landscapes with constant resources and produced an average of 22% more adults when patch configuration was dynamic. Abundance of beetles outside of patches was time-dependent, exhibiting greater abundance over time in landscapes with constant resources compared to those with diminishing resources. The effect of high connectivity on adults occurring in the matrix was 22% greater for landscapes with static patch configuration relative to dynamic configuration (Table 2, Fig. 3).

Dynamics of subadult beetles were affected by interactions of time with each of the experimental variables, and by interactions of resource×patch configuration and connectivity×patch configuration (Table 2). Larval and pupal abundance in patches declined over time, with more rapid declines for populations characterized by diminishing (versus constant) resource levels, high (versus low) connectivity, or static (versus dynamic) patch configuration (Table 2). A positive effect of dynamic patch configuration on larval and pupal abundance was evident only when connectivity was low (Table 2), and resulted in 17% more larvae and 86% more pupae than in landscapes with static configuration. Larval abundance was positively affected by a dynamic configuration of patches (10% increase relative to static configuration), but only when resources were constant (Table 2).

**Figure 4:**
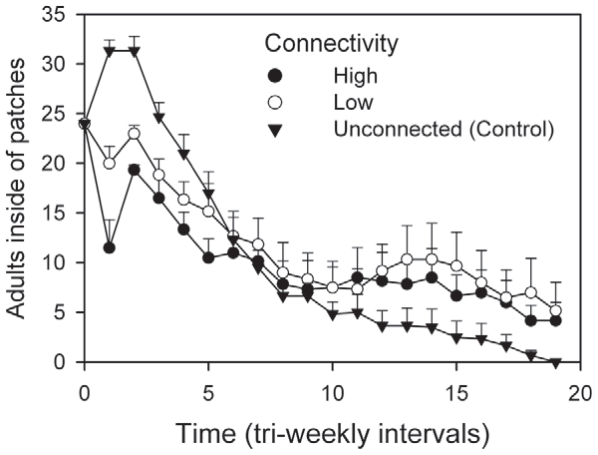
Intermediate levels of connectivity resulted in the greatest abundance of adults in patches. Landscapes with no connectivity resulted in an early increase of adults, followed by relatively rapid declines to extinction. Landscapes with patches that had some connectivity experienced early declines followed by stability over the last half of the study, with greatest abundance for landscapes with low connectivity. Values are means (+SE) of replicates.

For landscapes with static patch configuration, including control landscapes, significant interactions of time×resource and time×connectivity were evident (Table 3). The average number of live adults in patches declined over time, and declines were more rapid for metapopulations with diminishing resource patches. Landscapes with connected patches tended to have more adult beetles in patches relative to landscapes with no connectivity (Table 3). The interaction effect between time and connectivity indicated a temporary increase in the number of adults in populations with unconnected patches, followed by dramatic declines to extinction (Table 3). Populations with connected patches exhibited neither rapid increases nor crashes and stabilized around carrying capacity (Fig. 4). Results for larval abundance were similar to those for adults and are not presented here.

Poisson GLMM revealed that pupal abundance at time t declined with increasing adult abundance at time t-1 for both treatment and control landscapes (Table 4). Negative density dependence of pupae was observed in all landscape treatments with constant resources, with one exception (Table 4). In contrast, negative density dependence was evident in only two landscape treatments with diminishing resources, and both of these instances involved static patch configuration (Table 4). Negative density dependence was 1.7 times greater in landscapes with low (versus high) connectivity (Table 4).

**Table 4:** Mean pupal abundance, strength of negative density dependence (β) on adults and 95% credible intervals.

Treatment #		Factors		Mean # of Pupae	DensityDependence
	Connectivity	Resource	Configuration		(β and 95% CI)
1	High	Constant	Static	2.52	−0.04 (−0.10–0.01)^a^
2	High	Constant	Dynamic	2.58	0.02 (−0.04–0.07)
3	High	Diminish	Static	0.36	−0.12 (−0.25–−0.01)*
4	High	Diminish	Dynamic	0.24	−0.05 (−0.20–0.10)
5	Low	Constant	Static	2.17	−0.06 (−0.11–−0.02)*
6	Low	Constant	Dynamic	3.80	−0.05 (−0.08–−0.02)*
7	Low	Diminish	Static	0.32	−0.30 (−0.55–−0.10)*
8	Low	Diminish	Dynamic	0.73	0.09 (−0.09–0.28)
Control 1	None	Constant	Static	1.22	−0.11 (−0.20–−0.02)*
Control 2	None	Diminish	Static	0.15	−0.06 (−0.23–0.10)

For interpretation of *, see footnote for Table 2.

a The fraction of the posterior distribution more extreme than zero, *f* = 0.05.

### Metapopulation stability

Metapopulations with constant resources (*β* = −0.12, P = 0.0008) or low connectivity (*β* = 0.08, P = 0.01) produced lower fluctuations in amplitude than those with diminishing resources or high connectivity, respectively. Moreover, the effect of low connectivity on fluctuations tended to be greater for landscapes with constant resources (*β* = −0.06, P = 0.14). Mean fluctuations of metapopulations in landscapes with static versus dynamic patch configurations did not differ (*β* = 0.02, P = 0.49). For all landscapes, habitat patches (0.41) always fluctuated less than marginal habitats (3.34) (F = 41.64, P≪0.0001).

Patterns of fluctuations at the subpopulation level (habitat and marginal habitat) differed dramatically from those observed for entire metapopulations. Fluctuation in amplitude was 10 times lower (*β* = −3.11, P<0.0001) for habitat patches experiencing static (0.34) versus dynamic (3.4) configuration, but did not differ for other main effects. Fluctuations of subpopulations in habitat patches were influenced by the interaction of resource and configuration (*β* = −0.18, P<0.0002) as well as connectivity and configuration (*β* = 0.14, P = 0.003). Effects of configuration dominated in both instances, with greatest fluctuations in habitat patches in dynamic landscapes. When resource levels were constant, the effect of dynamic patch configuration on fluctuations in habitats was 1.52 times greater than when resource levels were diminishing. Similarly, when connectivity of habitat patches was low, the effect of dynamic configuration on fluctuations was 1.34 times greater than when connectivity was high. Fluctuations in marginal habitats were lower in landscapes with low patch connectivity (*β* = −1.61, P = 0.005) and a static configuration of patches (*β* = −6.06, P<0.0001), but no interaction effects were significant.

## Discussion

Beetles in our constructed landscapes met the four criteria for a metapopulation [36]. Namely, suitable habitat was configured in discrete patches (Fig. 1), local populations experienced measurable rates of extinction (Table 1), local population dynamics were not completely synchronized, and dispersing individuals linked the local populations. Regarding the latter point, dispersal was rare but sufficient to link local populations, averaging 0.4 (low connectivity) and 1.4 (high connectivity) individuals per generation.

The most noteworthy results from our study were the unexpected effects of patch connectivity and configuration on beetle metapopulations, and the manner in which effects interacted to influence abundance and stability. Dispersing *T. castaneum* have shorter developmental times and greater fecundity than non-dispersers [37]. Therefore, we expected metapopulations of beetles in highly connected landscapes to exhibit greater abundance and persistence. Instead, high connectivity led to greater mortality of dispersing beetles and produced an anti-rescue effect [38] that resulted in lower metapopulation size. Average adult mortality in the matrix was nearly 2.5 fold greater in landscapes with high (1.64) versus low (0.69) connectivity, corresponding to a nearly 3-fold increase in the magnitude of dispersers in landscapes with high connectivity. Consequently, landscapes with low connectivity had higher recruitment to and natality in habitable patches, and hence greater metapopulation abundance than landscapes with high connectivity.

Temporal dynamics of resource patches simulated apparent habitat loss and re-creation, and we expected survival and persistence of metapopulations to be impacted negatively [39]. Instead, landscapes with dynamic patch configuration supported larger metapopulations than landscapes with static patches. Landscapes with dynamic patches supported greater numbers of both adults and subadults, owing to increased survival associated with lower cannibalism. Preliminary trials revealed that the mature larvae and pupae that previously had been nourished by resources in a habitat patch managed to develop and metamorphose to adults in their newly occupied marginal-habitat patch. Adult beetles occupying marginal-habitat patches in our dynamic landscapes were motivated to disperse and quickly occupy newly created habitat patches. Their dispersal to habitat patches apparently reduced cannibalistic activity in marginal-habitat patches. At the onset of each tri-weekly period throughout the study, newly created habitat patches (i.e. transformed marginal habitats) in our dynamic landscapes had negligibly low adult and egg density, whereas the habitat patches in static landscapes retained adults and eggs at greater densities. Low initial densities, combined with gradual recruitment of dispersing adults, likely facilitated enhanced adult fecundity in newly formed habitat patches of dynamic landscapes [40], [41]. Thus, dynamic landscapes contained patches that changed states between habitat and marginal habitat, effectively providing refugia for juveniles, pupae and callows by releasing them from cannibalism [42]. Vuilleumier and coworkers [24] concluded that any patch in a dynamic landscape that provides refugia can serve as source of colonists for habitats recovering from disturbance, thereby increasing metapopulation persistence.

Metapopulations with constant resource levels and low connectivity exhibited the greatest levels of density dependence. Consistent with Desharnais and Liu [43], these metapopulations also exhibited the greatest stability. Strong intraspecific competition at high population densities can reduce population variability and local extinction probabilities [44], [45], as shown experimentally in our study and in rock pool *Daphnia* populations [46], [47].

In our study, reduced patch connectivity increased the difference in colonization rate between dynamic and static landscapes by 3–30-fold. Connectivity had no effect on extinction rate, but high connectivity resulted in more adults, and more adult mortality, in the matrix for landscapes with static patch configuration. Thus, the net effect of low connectivity was increased patch occupancy and abundance for metapopulations in dynamic landscapes. Despite fluctuations of habitat subpopulations that were 10 times greater for dynamic landscapes, metapopulations in dynamic landscapes displayed stability comparable to those in static landscapes. Asynchrony in the dynamics of subpopulations likely contributed to this effect, which was especially notable when connectivity was low. Following patch destruction, marginal habitats served as more effective refugia for mature larvae, pupae and callow when connectivity was low; a similar effect on recruitment was shown in a coral reef fish (*Dascyllus flavicaudus*) [48]. In contrast, dynamics of subpopulations in static landscapes or with higher connectivity experienced greater synchrony; habitat patches varied together at comparable adult densities, and marginal habitat patches were occupied only at low levels throughout the study. Our results agree with those of Dey and Joshi [17], who attributed higher stability in less-well-connected fruit fly metapopulations to asynchrony in neighboring subpopulations. Thus, increasing connectivity may be counter-productive for some species.

Other investigators have noted potentially deleterious effects of enhanced connectivity. Hess [9] predicted an anti-rescue effect with increasing connectivity among subpopulations owing to increased predation, spread of infectious disease, or other factors that enhance the local extinction rate relative to recolonization. Molofsky and Ferdy [18] found a nonlinear relation between migration rates and persistence time in metapopulations of the herb (*Cardamine pensylvanica*) and observed increased extinction due to increased connectivity when all subpopulations in a metapopulation fluctuated in synchrony and consequently experienced simultaneous decline. Conflicting results regarding the impact of connectivity on metapopulation dynamics suggest that predicting the effects of connectivity between habitat patches requires consideration of the dispersal ability of a species and how behaviors modified by landscape heterogeneity influence survival or reproduction [17], [49]. In addition to dispersal and behavior, our findings indicate that changes in habitat configuration can interact with patch connectivity to influence metapopulation dynamics. Specifically, lower connectivity may be advantageous in dynamic landscapes if it reduces competition and improves juvenile survival via creation of refugia for critical life stages.

Answering questions about optimal connectivity will likely require an understanding of the biology of a target species and the resource requirements for each of its life stages. Our findings suggest that questions of connectivity should simultaneously consider the temporal dynamics of preferred and marginal habitats. Clearly, additional empirical studies are needed to explore the impact of patch turn-over rates and habitat complexity on metapopulations residing in spatially and temporally varying landscapes.

## Supporting Information

Appendix S1 **WinBUGS script for specifying Poisson mixed effect regression model for the response variable to estimate model parameters.** The modelled data are C[i,j], which represent counts of adult beetles in landscape *j* at time *i*. Comments follow a #.
(DOC)
