# Supplementary material for: Experimental Beetle Metapopulations Respond Positively to Dynamic Landscapes and Reduced Connectivity
Source: PLoS One. 2012 Apr 3;7(4):e34518. doi: 10.1371/journal.pone.0034518 (PMC3317989; doi:10.1371/journal.pone.0034518)
Supplement: Appendix S1 — WinBUGS script for specifying Poisson mixed effect regression model for the response variable to estimate model parameters. The modelled data are C[i,j], which represent counts of adult beetles in landscape j at time i. Comments follow a #. (DOC) [file pone.0034518.s001.doc]

**Appendix S1.** WinBUGS script for specifying Poisson mixed effect regression model for the response variable to estimate model parameters. The modelled data are C[i,j], that represent adult beetle count in landscape *j* at time *i*. Comments follow a #.

model {

# Priors

mu ~ dnorm(0.0,1.0E-4) # Grand mean

beta0 ~ dnorm(0, 0.01) # Overall linear time trend

beta1 ~ dnorm(0.0,1.0E-4) # For Resource

beta2 ~ dnorm(0.0,1.0E-4) # For Connectivity

beta3 ~ dnorm(0.0,1.0E-4) # For Patch Dynamics

beta4 ~ dnorm(0.0,1.0E-4) # For Resource x Connectivity

beta5 ~ dnorm(0.0,1.0E-4) # For Resource x Patch Dynamics

beta6 ~ dnorm(0.0,1.0E-4) # For Connectivity x Patch Dynamics

beta7 ~ dnorm(0.0,1.0E-4) # For Resource x Time

beta8 ~ dnorm(0.0,1.0E-4) # For Connectivity x Time

beta9 ~ dnorm(0.0,1.0E-4) # For Patch Dynamics x Time

for (j in 1:nReplicn){

alpha [j] ~ dnorm(0, tau.alpha) # Random-effects distribution 1 (8*3 = 24 subjects)

}

tau.alpha <- 1/ (sd.alpha * sd.alpha)

sd.alpha ~ dunif(0, 1) # Hyperprior for dispersion hyperparam1

for (i in 1:nTime){ # Random-effects distribution 2 (23 time steps)

eps[i] ~ dnorm(0, tau.eps)

}

tau.eps <- 1/ (sd.eps * sd.eps)

sd.eps ~ dunif(0, 1) # Hyperprior for dispersion hyperparam 2

# Likelihood

for (i in 1:nTime){

for (j in 1:nReplicn){

C[i,j] ~ dpois(lambda[i,j]) # 1. Distribution for random part

lambda[i,j] <- exp(log.lambda[i,j]) # 2. Link function

log.lambda[i,j] <- mu + beta0 * CTime[i] + beta1 * (Resource[j]) + beta2 * (Connect[j]) + beta3 * (Config[j]) + beta4 *(Resource[j]) * (Connect[j]) + beta5 * (Resource[j]) * (Config[j]) + beta6 * (Connect[j]) * (Config[j]) + beta7 *(Resource[j]) * (CTime[i]) + beta8 * (Connect[j]) * (CTime[i]) + beta9 * (Config[j])* (CTime[i]) + alpha[j] + eps[i] # 3. Linear predictor

} # j

} # i

} # model
